# A Heroic Fireman

**Published:** 1887-08-20

**Authors:** Thomas Ryan

**Affiliations:** Secretary. St. Mary's Hospital


### THE EDITOR'S LETTER-BOX.

[Our Correspondents are reminded that prolixity is a great bar to publication, and that brevity of style and conciseness of statement greatly facilitate early publication.]

### A HEROIC FIREMAN.

SIR,—The two firemen belonging to the Hermitage Street Station who were on the second-floor of Whiteley's establishment on Saturday evening last, directing the hose at the fire when the roof fell in, severely injuring them, were admitted to this hospital the same night. The story of their escape has not yet appeared, but it is so interesting, and the bravery of one of the men was so conspicuous, that it should not be allowed to pass unrecorded.

It appears that the Hermitage Street engine, which was the first to arrive on the scene, and which seems to have enjoyed an unenviable monopoly of disaster, reached the fire at about 7.30. As soon as possible two firemen—the two who are now among our in-patients—named respectively James Brown and Ambrose Lester, were on the landing of the second-floor with the hose, outside the room in which the fire was raging, directing the water through the doorway on to the flames. Then came the disaster which turned the fortunes of that most eventful night—for up to that moment there seemed every prospect that the water would gain the mastery—and which was the cause of the only loss of life that occurred, and of the most serious of the accidents. They had not been on the landing more than six or seven minutes when a terrible crash was heard, and at the same time a mass of fire belched forth through the doorway on to the landing where the devoted firemen stood, completely enveloping them in flames. The roof of the room had fallen in, and the flames which filled it were forced in one huge column of fire through the doorway. Blinded by the blaze, and before they had time to retreat, or even to think, they were hurled by the masses of falling roof down the flight of stairs at the top of which they had stood, on to the next landing. Bruised and bleeding, with his face and hands scorched and burnt, Lester was still not unconscious, and was able to find his way down the remaining stairs into the street. On reaching the open air he turned round to find his comrade, who he expected was following, when to his horror he discovered he was alone. Though so injured and shaken that he was scarcely able to crawl, Lester at once returned, and at the risk of his life made his way back to the landing he had just quitted. Although only a few seconds had elapsed he found the staircase ablaze, and his comrade lying on the floor in the midst of a heap of *débris*, and nearly surrounded by the flames. With much difficulty he managed to rouse him and get him on his feet, and then down the stairs into the street. "Ah! sir," said Brown to me this afternoon, "if my mate hadn't come back and got me out of that place, I shouldn't have seen daylight again, or be lying in this hospital now a-telling you the tale."

Such is the story, unskilfully told, of the hairbreadth escapes of Lester and Brown. If ever a deed of calm, bold pluck was done, it was the return of Lester for his fallen comrade. Not less truly than the remnant of the Light Brigade did he and his mate come

"Back from the jaws of Death,

Back from the mouth of Hell."

And when we remember that the "pomp and circumstance of war" was not there, nor the cheer of comrades, nor the excitement of battle, I make bold to say that the fireman is not less a hero than the soldier.—Yours faithfully, THOMAS RYAN, Secretary.

St. Mary's Hospital, 12th August, 1887.

